# Artificial intelligence and radiomics for radiation oncology

**DOI:** 10.1007/s00066-020-01676-y

**Published:** 2020-09-17

**Authors:** Martin Kocher

**Affiliations:** 1 Department of Stereotaxy and Functional Neurosurgery, University Hospital of Cologne, Cologne, Germany; 2 Institute of Neuroscience and Medicine, Research Center Juelich, Juelich, Germany

Human intelligent behavior includes the classification of sensory information based on previous experiences, the planning of complex actions, and the prediction of future events based on internal models of the world. Although these skills have developed in the broad natural human environment, they can be adapted to special, restricted situations such as the diagnosis and treatment of diseases. Through years of training, physicians thus learn to interpret clinical signs, laboratory values, and medical images in order to come to a probable diagnosis, to plan a useful treatment, and to estimate the outcome.

Technical developments in recent years support the view that many of these steps can be performed by computer programs that are based on models which have been trained on digitalized medical data. The largest amount of data is generated by medical imaging and it thus comes without surprise that the automated detection and classification of radiological findings is one of the fastest growing fields in this research area. However, due the enormous importance of medical imaging for radiotherapy planning, radiation delivery, and outcome assessment, radiation oncology is especially suited for the application of these automated methods.

In radiation oncology, the first step is usually to identify the tumorous tissue to be irradiated, a procedure which is called image segmentation. In computerized image analysis methods, segmentation is based on the detection of a high-dimensional set of imaging features (sometimes also called “texture”) which are present in the tumor but absent in the normal tissue. As a multitude of features are usually termed “omics” in the biomedical sciences, the term “radiomics” or “radiogenomics” has been adopted for this kind of analysis. Once the typical features have been identified, radiomics can be used to detect tumor areas suitable for dose escalation, to correlate molecular markers with imaging features, to predict response and oncological outcome, and to differentiate treatment-related tissue changes from tumor recurrences.

A multitude of computerized mathematical and statistical methods stemming from the modern era of computational bioinformatics and machine learning exist that make these analyses possible. This special issue intends to inform the interested physician and physicist in radiation oncology about the general principles and current achievements of these methods in a selection of tumors usually treated by radiation therapy.

